# Making mathematics meaningful for freshmen students: investigating students’ preferences of pre-class videos

**DOI:** 10.1186/s41039-015-0026-9

**Published:** 2016-01-07

**Authors:** Rim Gouia, Cindy Gunn

**Affiliations:** 1 Department of Mathematics and Statistics, American University of Sharjah, P.O. Box 26 666, Sharjah, United Arab Emirates; 2 Faculty Development Center, American University of Sharjah, P.O. Box 26 666, Sharjah, United Arab Emirates

**Keywords:** Flipped classroom, Pre-class videos, University undergraduate Mathematics classes

## Abstract

Engaging students in university mathematics classes can be a challenge for professors. One pedagogical technique is the use of pre-class videos in a flipped classroom. The students are exposed to the concepts and theories before attending class so that class time can be devoted to interacting with the content to better understand it. Most of the research into the flipped classroom shows that the students generally like the idea and feel they benefit from the approach; but to date, there is no conclusive research showing that students’ improve their grades. This research is a precursor to a larger study on the flipped classroom in university mathematics classes and investigates the types of videos undergraduate students prefer to help guide the development of a pre-class videos library. Eight-one students in three university mathematics classes in a private university in the United Arab Emirates were involved in the study.

**Electronic supplementary material:**

The online version of this article (doi:10.1186/s41039-015-0026-9) contains supplementary material, which is available to authorized users.

## Background information

As Bishop and Verleger (2013) note, “there is a considerable amount of buzz in academic circles at all levels, focused around the flipped classroom … [but] there is a lack of consensus on what exactly the flipped classroom is”. An organization devoted to the flipped classroom, the flipped learning network (http://flippedclassroom.org/) offers the following definition of flipped learning:

- A pedagogical approach in which direct instruction moves from the group learning space to the individual learning space, and the resulting group space is transformed into a dynamic, interactive learning environment where the educator guides students as they apply concepts and engage creatively in the subject matter.

As noted by Hamdan et al. 2013, “there is an established body of research that supports … the shift from a teacher-centered to a student-centered approach to instruction: (2013, p. 6). In addition, flipped learning, by encouraging a shift to a learning paradigm in higher education and by moving direct instruction to the individual space, “addresses one challenge facing many instructors interested in creating dynamic learning environments: How to free up time during class” (Wallace et al. 2014, p. 254).

**Fig. 1:**
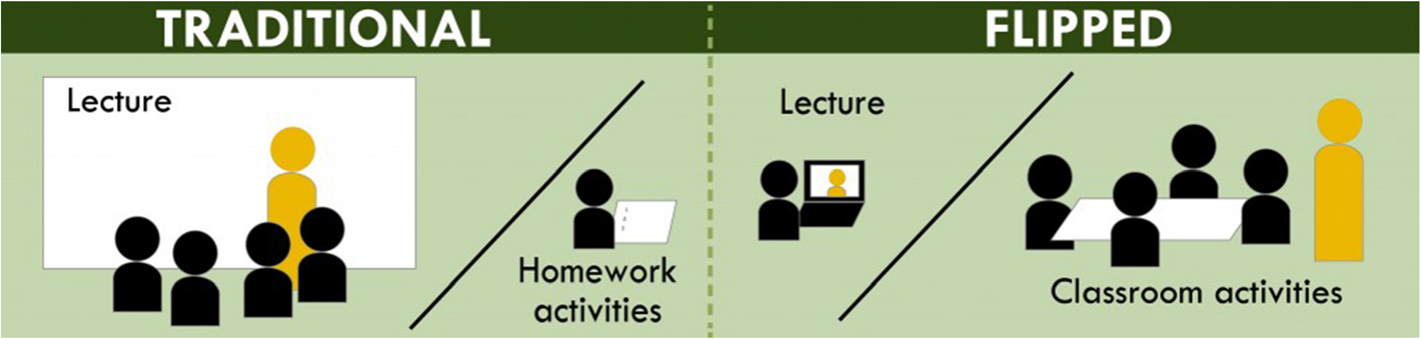
Traditional versus flipped classrooms (http://www.washington.edu/teaching/teaching-resources/engaging-students-in-learning/flipping-theclassroom/). The image depicts the movement of instruction from the group space to the individual space and resulting in changing classroom dynamics

Figure 1 depicts the movement of instruction from the group space to the individual space and resulting changing classroom dynamics.

The benefits of the flipped classroom to the students are numerous and encourage high-impact practices. These practices include, “students taking responsibility for their own learning, investing time and energy in practice, collaborating with classmates around challenging learning activities, receiving and responding to frequent and timely feedback from instructors, and seeking to connect their learning to real-life applications” (Kul et al., 2010, cited in Wallace et al. 2014, p. 254). For example, students can take responsibility for their own learning by accessing the content anytime, anywhere, on any device. In addition, students can pause and rewind and review the video, or sections of the video, as often as they like. Interaction and active learning opportunities are increased during class time, and all students, regardless of ability can be engaged and challenged to further their understanding of the material by their peers and their instructors.

The hallmark of the flipped classroom “consists of two parts: interactive group learning activities inside the classroom, and direct computer-based individual instruction outside the classroom” (Bishop and Verleger 2013). The use of pre-class videos is the most common technique associated with the computer-based instruction. In their study, Long et al. (2014) found that 78 % of the students agreed or strongly agreed that they prefer learning via videos. The students stated that the videos helped them understand the knowledge; they were easy to follow and were convenient to view. Murray et al. (2015) also found that students were generally positive about the flipped classroom particularly the convenience and the flexibility of the videos.

The types of videos vary from information displayed on the screen in bullet points with voice over narration to full instructor presence working at the board. The length of the videos also varies. Ferrer and García-Barrera (2014), suggest adhering to the segmentation principle and break up the information into smaller segments. They also discuss three personalization principles to consider when preparing the instructional videos. These include:

1. Informal language principle: it is better to use informal language (first and second person) than formal language.
2. Guide principle: incorporating characters on screen that fulfill a coaching role promoting learning.
3. Author visibility principle: when the author is an active and visible element in the video, personally involved in the narration, learning is more successful (p. 2610).

One of the main concerns about the flipped classroom is whether or not the students will do the pre-class preparation, whether they will watch the video or other online activities assigned by the teacher. Since the class time is generally devoted to group work or other tasks relying on the students having done the preparation work, this is a legitimate concern. However, in their ongoing large scale study with Engineering and Mathematics students, Lape et al. (2015) have found that the students report they do watch the video to prepare for the in-class activities.

### Flipped learning in mathematics classes

Mathematics can be a challenging subject for many students, and it can be difficult for teachers at all levels to help the students reach at their full potential. In higher education, math courses can be a stumbling block toward majoring in STEM fields. It is a required course for the engineering fields, but even some of those students struggle with understanding and applying the higher level concepts presented in a traditional lecture class. The flipped classroom allows students to apply what they have learned, usually in a group setting. The results, as demonstrated in two recent studies, can be better understanding of the material and higher grades. For example, McGivney-Burelle and Fei (2013) conducted a study with two calculus classes. One was taught in a traditional, lecture format and the other was in a flipped classroom. They found that the students in the flipped Calculus classroom received higher grades than the students in the traditional class. Syam (2014) also conducted a pilot study of the flipped classroom in a pre-calculus course with his students at Qatar University for 2 weeks to cover three sections from the syllabus. At the end of the trial period, students had to complete a quiz to assess their understanding of the material. Syam found that the results of this quiz were higher than the previous quizzes where students were not taught in a flipped classroom. In addition, a questionnaire was distributed to the students to probe student perceptions more deeply. The results suggest that the majority of students preferred the flipped classroom and would rather take future mathematics classes in this manner.

## Methods

Although not without its criticisms, there is enough evidence to suggest that students can benefit from the flipped classroom and that those who are involved in a flipped class enjoy it. Although there is general consensus in the literature that students strongly prefer the videos made by their professors, before investing a lot of time and energy into preparing the pre-class videos, the lead author wanted to find out the students’ attitudes towards the usefulness of the pre-class videos and the type of videos that they found the most useful. Thus, in this study, three videos from YouTube were used. The students were surveyed to identify their preferences to guide the development of a future video library prepared by the Professor.

The study was conducted in Spring 2015 with three Undergraduate Math classes at American University of Sharjah (AUS). AUS is a coeducational, private institution in Sharjah, United Arab Emirates. It has a student body of approximately 6000 students representing over 90 nationalities. The students come from different educational systems with different prior learning environments especially when it comes to mathematics. Permission to conduct the study with AUS students was granted by the AUS Internal Review Board. 

Eighty-one students, 46 females, and 35 males, in two sections of MTH 103, Calculus for Engineers, and one section of MTH 111, Mathematics for Architects participated in this study. MTH 103 is a fundamental course that builds the mathematical foundations to engineering and other scientific fields. This course covers limit of functions, differentiation, applications of derivatives, and theory of integration with applications. For many students, this course is a stumbling block toward majoring in STEM fields. MTH 111 introduces the topics of geometry and calculus needed for architecture, reviews trigonometry, areas and volumes of elementary geometric figures, and the analytic geometry of lines, planes and vectors in two and three dimensions.

### Data collection

In preparation for the flipped class, three videos that are freely available on YouTube were reviewed and chosen by the Professor. The length of the videos, the presentation style, and the level of detail in the explanation varied as shown in Table 1. These videos were chosen for their difference in presentation modes of the same topic. The students were given access to the three online videos through the AUS Course Management System.

**Table 1:** Description of videos

Video	Length	Presentation style	Explanation
A	16 min	Chalk board and teacher visible	Very detailed
B	11 min	Electronic screen, narrated, teacher not visible	Details available in a side bar
C	4 min	Pen, paper, hands, narrated, teacher not visible	Concept focus

The Professor informed the students that their next class would be conducted differently and explained the concept of the flipped classroom. She showed them where to find the videos on the course management system and told the students that she would prefer if they watched all three but were expected to watch at least one of the videos. The students were well aware that the Professor was not going to give a lecture on the Chain Rule and that they needed to watch the pre-class videos and be prepared to do some in-class exercises to better understand this rule.

Before starting the group exercises, the students filled out a short survey about the videos. The survey was designed on purpose to be short and clear to encourage the students to fill it out. They were under no obligation to do so and were not rewarded or punished for either participating or not participating. The survey was designed to gather demographic information and both quantitative and qualitative data. It can be found in Additional file 1.

## Results and discussion

One of the concerns expressed in the literature is whether or not the students will watch the pre-class videos. In spite of being well informed of the videos’ availability and how the class would be structured differently, a total of 43 % (37 % female and 51 % male) did not watch the videos. Reasons for not watching the videos included forgetting about the assignment and running out of time because of other homework assignments for other classes. One female student noted that she did not watch the videos because she felt she already know the information on the videos, and one male student did not watch because he “thought it will be explained in class and I understand from my professor perfectly”.

**Fig. 2:**
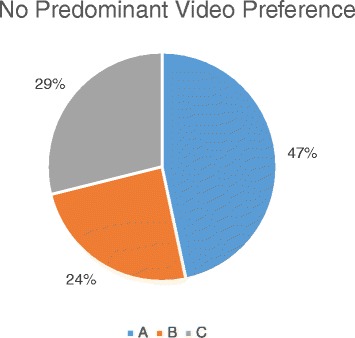
Video preference for both classes for both genders

Of the 57 % of the students who did watch the videos, there was no predominant video preference as shown in Fig. 2. In addition, there was no statistical correlation between the students’ majors (engineers and architects) and their general video preference. However, the highest percentage is for the longer video with more details and the ability to see the teacher.

**Fig. 3:**
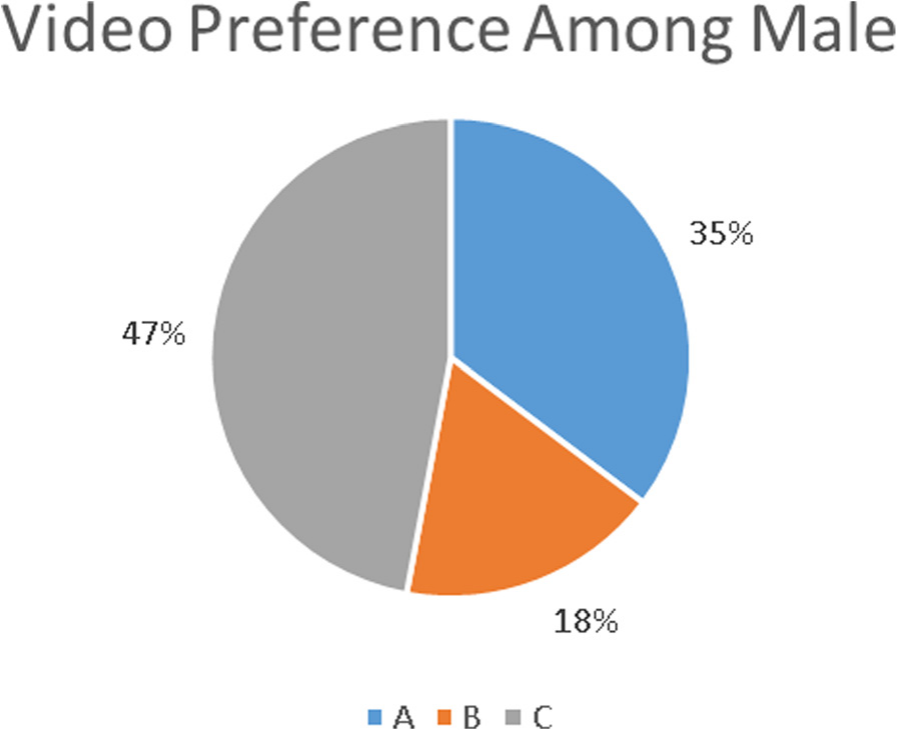
Video preference among males

**Fig. 4:**
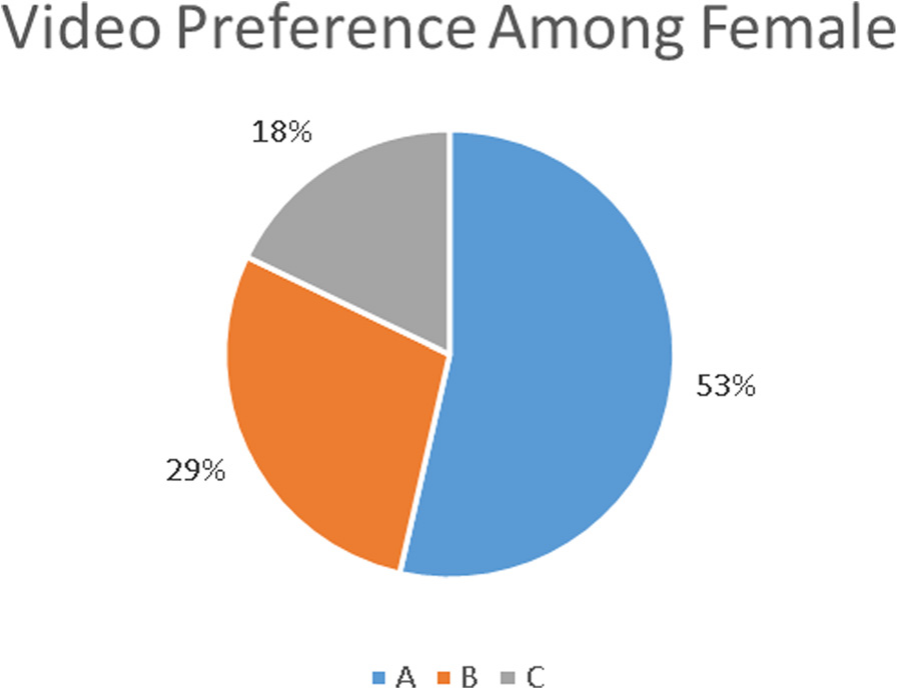
Video preference among females

There were, however, some gender differences in video preference. A higher percentage of the females viewed the videos for a total of 57 versus 49 % of the males. As demonstrated in Figs. 3 and 4, the females had a preference for the longer video (A) compared to the males who showed a preference for the shorter video (C).

Some of the students watched all three videos, while others watched only one. The reasons for watching just one, for both males and females, were mainly centered on the lack of time. Comments from the students who watched all three videos explaining their preferences included:

- I am a visual learner, so to understand very well, I must see examples: Also, watching video B after video C was useful because I had already learned the basic rules from video C. When it came to video A, I was bored and did not need more information. Video B was also effective because the basic rules (3) of the chin rule were always available on the right hand side easy for me to relate back to. (Watched all 3)
- Shorter videos are easier to focus on.
- The videos overall were useful. They explained the chain rule in the same way over a long period of time which I preferred. All three were useful.

### Students’ attitudes towards the use of the pre-class videos

Overall, the students were positive about the videos and their usefulness. There were, however, two students, one female, and one male, who expressed their dissatisfaction with the videos. The female noted that: “I rather have my lecture in class with the teachers teaching style that I am comfortable with. Prefer videos after class than before”. The male student wrote, “The flipped class is a bad idea. Normal classes are better.” Thus, we can understand his point, but we do not know the reason why he does not like the flipped classroom. This comment, however, can be seen to be consistent with Talbert’s observation that in his mathematics classes, “many students in flipped classrooms are rebelling because they want professors to lecture to them and tell them exactly how to earn a good grade” (2015 p. 15). He recommends, acknowledging in class that some students will feel anxious or uncertain about learning in a new, unfamiliar way, and to take time to explain how they may benefit from the flipped classroom. Both negative comments underscore the importance of uncovering students’ preferences to best help them learn. Further, as Barret notes, “the techniques all share the same underlying imperative: Students cannot passively receive material in class, which is one reason some students dislike flipping” (Berret 2014, p.2).

The positive comments from the males who watched at least one of the videos included the following:

- It really helped to understand the material better
- The videos were a great way to understand the material at home.
- Nothing other than they are “very helpful”. Keep posting more.
- I like this idea since it is very convenient and enables better understanding because you get to watch it your own pace.

Positive comments from females who watched at least one of the videos included:

- We should have more of these videos and practice more during class. Thank you.
- It’s an effective way of learning. I wish there was videos to watch for all my courses and subjects and I wish if we took more than the Chain Rule.

### Observations from the professor

In the flipped class, out of the 50-min-allocated class time, 35 min were dedicated to student active learning. The students were given a set of problems to solve starting with very straightforward ones and gradually getting more challenging. As the students were working on problems and interacting with each other, the professor noticed that they were motivated, sharing their knowledge, and explaining concepts to their peers. Because the concepts, the theorems, and also the conditions of applying these mathematical notions were covered in the pre-class videos, she was able to freely move from one group to another, answering questions, and correcting solutions. She was able to help them at the right time and meet the needs of all students from all ability levels. For example, the struggling learners needed more extra help than the rest of the class. Because some of them are lacking very basic mathematics due to the fact that they are coming from different educational systems with different prior learning environments, they needed one-on-one time to understand the basic concepts and fill their knowledge gaps.

The middle level students were able to solve the problems and often benefited from the interactions with the high achievers and anchored their knowledge as they argued with their peers or assisted the lower level students. The advanced students asked for higher level thinking problems. They quickly solved the easy questions and had time to think about the in-depth problems, and this allowed them the opportunity to reach their full potential. They were not limited or held back with basic explanations or easy question from a low-level student.

The opportunity to have one-on-one conversations with many students during class time was a real benefit of the flipped class. The professor had conversations with students that she had never talked to before because they were too shy to talk in front of their peers or they are afraid of making mistakes. During this experience, they were able to ask questions, clarify misconceptions, and be an active participant in the classroom.

## Conclusion

In spite of the fact that some students did not watch the videos, the overall purpose of this small scale research was achieved. That is, the professor was able to make the mathematics class more meaningful for the students with the use of the pre-class videos. She was not constrained to delivering a “one standard lecture” to fit all. Because of the nature of the group work assignments, even the students who did not watch the videos had the opportunity to interact with the content and learn from each other. All the students, regardless of ability level were moving in the right direction with each student progressing towards his/her full potential at his/her own pace.

The positive feedback from the students and the professor’s observation of the benefits to the students is the impetus for the next phase of implementing the flipped classroom. Although there was no clear preference for the type of video, long, short, detailed, etc., the professor is now ready to prepare her own pre-class videos and continue with the flipped classroom to make her classroom a more enriching, rewarding learning experience for the students. Her efforts are not lost on the students, as one student noted: “Thank you Prof. You are the only one who is helping us to improve.”

## Additional file 1

Additional file 1: **Student survey**. (DOC 26 kb)
