# Supplementary material for: Making mathematics meaningful for freshmen students: investigating students’ preferences of pre-class videos
Source: Res Pract Technol Enhanc Learn. 2016 Jan 7;11(1):2. doi: 10.1186/s41039-015-0026-9 (PMC6302848; doi:10.1186/s41039-015-0026-9)
Supplement: Supplementary file 1 — Student survey. (DOC 26 kb) [file 41039_2015_26_MOESM1_ESM.doc]

Appendix 1: Student Survey

Dear Student!

The following survey aims to collect information related to the pre-class videos in the flipped classroom. You are kindly requested to contribute to the development of courses offered by the Faculty by giving your opinion through filling in the questionnaire. It should not take you more than a couple of minutes to fill.

Thank in advance for your assistance.

Gender: Female ☐ Male ☐ Major: ______

Did you watch the videos? Yes ☐ No ☐

• If No, why not?

• If Yes, did you watch the videos to:

☐ Better understand the material

☐ Improve your grades

☐ Do the assigned homework

Other reason (please list)

Which video(s) did you watch? A ☐ B ☐ C ☐

Why?

Which one did you like the most? A ☐ B ☐ C ☐

What did you like about it?

Do you have any other comments about the videos?

Thank you.
